# High‐speed video and plant ultrastructure define mechanisms of gametophyte dispersal

**DOI:** 10.1002/aps3.11463

**Published:** 2022-04-20

**Authors:** Nora Mitchell, Nancy P. Piatczyc, Darren D. Wang, Joan Edwards

**Affiliations:** ^1^ Department of Biology University of Wisconsin–Eau Claire Eau Claire 54701 Wisconsin USA; ^2^ Biology Department Williams College, Williamstown Massachusetts 01267 USA

**Keywords:** biomimetics, gemmae, high‐speed video, pollination, spores

## Abstract

Dispersal of gametophytes is critical for land plant survivorship and reproduction. It defines potential colonization and geographical distribution as well as genetic mixing and evolution. C. T. Ingold's classic works on *Spore Discharge in Land Plants* and *Spore Liberation* review mechanisms for spore release and dispersal based on real‐time observations, basic histology, and light microscopy. Many mechanisms underlying spore liberation are explosive and have evolved independently multiple times. These mechanisms involve physiological processes such as water gain and loss, coupled with structural features using different plant tissues. Here we review how high‐speed video and analyses of ultrastructure have defined new biomechanical mechanisms for the dispersal of gametophytes through the dissemination of haploid diaspores, including spores, pollen, and asexual reproductive propagules. This comparative review highlights the diversity and importance of rapid movements in plants for dispersing gametophytes and considerations for using combinations of high‐speed video methods and microscopic techniques to understand these dispersal movements. A deeper understanding of these mechanisms is crucial not only for understanding gametophyte ecology but also for applied engineering and biomimetic applications used in human technologies.

Plants often rely on dispersal of the gametophytic stage for spread to new areas and exchange of genetic material, and in fact all major categories of land plants have some form of haploid diaspore or propagule. Here we use the term “gametophyte” to refer to either the gametophyte generation or the single‐celled spores that give rise to the multicellular gametophyte. The nature of the gametophyte varies in terms of being asexual or sexual and whether the dispersal agent is the single‐celled spore or the entire gametophyte generation. For instance, gemmae are asexual propagules of gametophytic tissue that are used as haploid dispersal agents by various seedless plants, such as in the splash cups of the liverworts *Marchantia* L. (Marchantiaceae) and *Lunularia* Adans. (Lunulariaceae) and the fern genus *Vittaria* Sm. (Pteridaceae) (Brodie, 1951; Emigh and Farrar, 1977; Equihua, 1987; Figure 1B).

**Figure 1:**
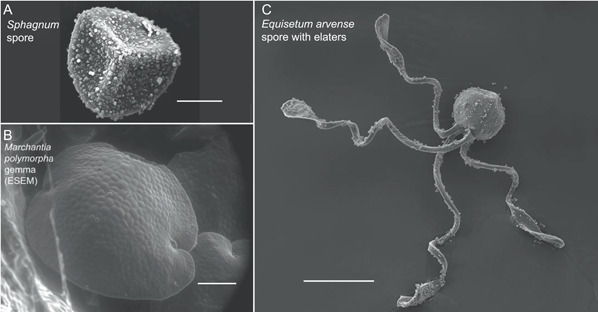
SEM images of example diaspores in non‐seed plants. (A) *Sphagnum* sp. spore. (B) *Marchantia polymorpha* gemma taken using environmental SEM, which allowed the sample to be fully hydrated. (C) *Equisetum arvense* spore with elaters. For these images, the spores in A and C were placed on double‐sided tape with carbon (Electron Microscopy Sciences, Hatfield, Pennsylvannia, USA) affixed to SEM stubs, sputter coated with gold/palladium, and viewed on an FEI Quanta 400 (FEI, Hillsboro, Oregon, USA). For the environmental scanning electron image of the *Marchantia* gemma, the fully hydrated gemmae in gemmae cups were placed on the cold stage at 5°C with no additional preparation and viewed on the FEI Quanta 400. Scale bars in (A) 10 μm, (B) 100 μm, and (C) 50 μm.

In non‐seed plants, the singled‐celled spores, which germinate into gametophytes, are generally the only diaspores that facilitate long‐distance dispersal. In nonvascular plants (“bryophytes”) such as mosses and liverworts, spores are produced in the capsule structure of the sporophyte generation and rely on this capsule for spore dispersal (e.g., Whitaker and Edwards, 2010; Figure 1A). For seedless vascular plants (i.e., lycophytes and monilophytes), haploid spores disperse (sometimes catapulted by sporangia, the spore‐bearing organs of the sporophyte generation), germinate, and develop into independent gametophytes from which the sporophyte generation emerges.

Pollen grains, which contain the immature male gametophyte, are the only gametophytic dispersal agent in seed plants (both gymnosperms and angiosperms) and are dispersed via numerous abiotic and biotic agents (Figure 2). Finally, there are also sporophytic diaspore units, namely seeds (which act as the main dispersal agent in seed plants), as well as various asexual sporophytic bulbils in ferns and lycophytes.

**Figure 2:**
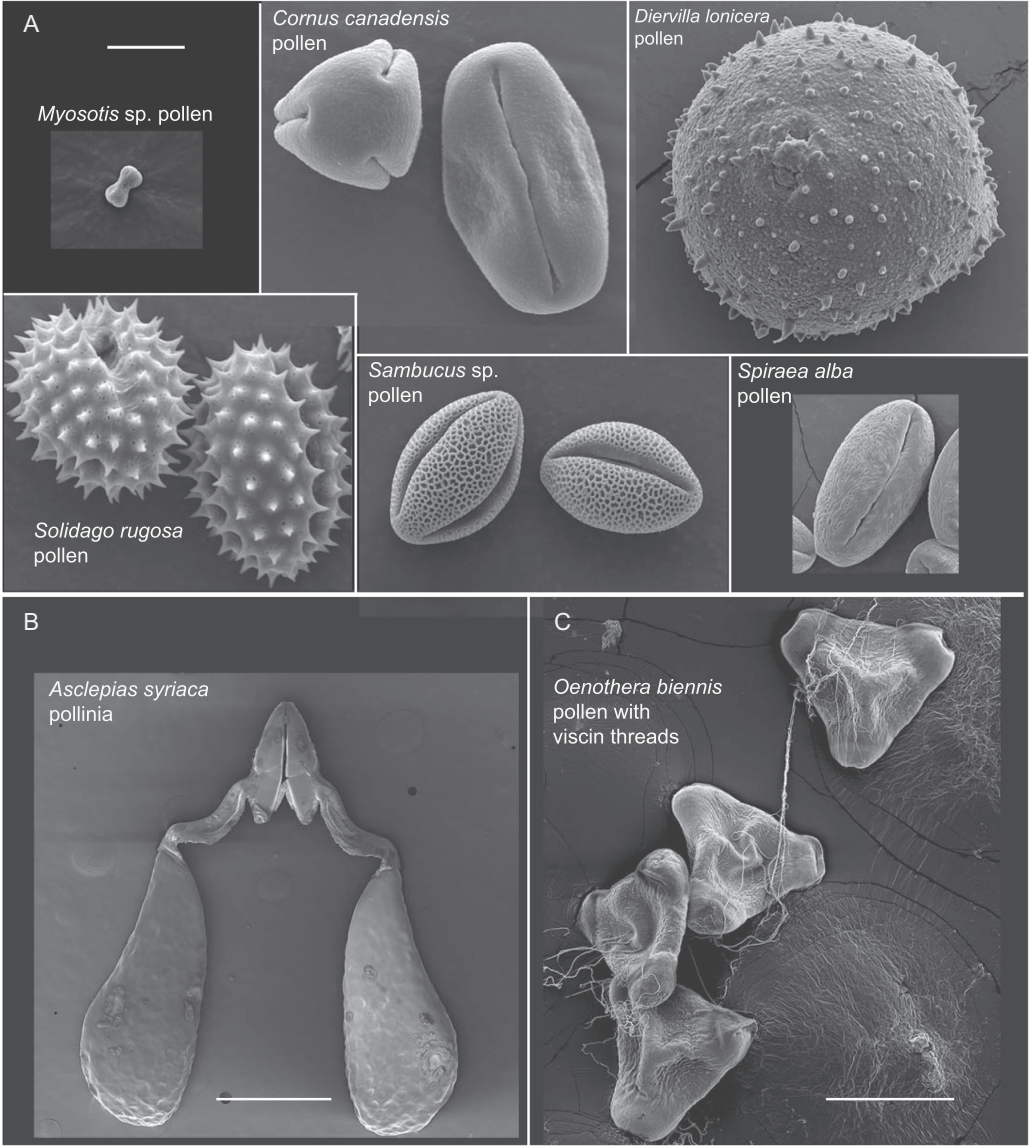
SEM images of pollen grains and pollinia show variation in size, shape, and ornamentation of dispersal units. (A) SEM of pollen grains taken separately but depicted at the same scale to illustrate variation in size, which can affect dispersal (e.g., terminal velocity). The pollinia of (B) *Asclepias syriac*a and (C) viscin threads linking pollen of *Oenothera biennis* show that pollen can be grouped into larger units for dispersal. For these images, the pollen or pollinia were placed on double‐sided tape with carbon (Electron Microscopy Sciences) and affixed to SEM stubs. *Myosotis*, *Cornus*, *Solidago*, and *Sambucus* specimens were sputter coated with gold/palladium and viewed on an FEI Quanta 400. *Diervilla*, *Spiraea*, *Asclepias*, and *Oenothera* were sputter coated with platinum on a Quorum Q150T ES Plus (Quorum Technologies, Laughton, United Kingdom) and viewed on a Thermo Scientific Quattro S (Thermo Scientific, Waltham, Massachusetts, USA). Scale bars in (A) 10 μm, (B) 0.5 mm, and (C) 100 μm.

Dispersal of these various gametophytes is essential in determining the geographic distribution of plants. This is especially true for non‐seed plants; for instance, the distribution of bryophytes and ferns is influenced by both local and long‐distance dispersal, resulting in most families and genera having broad, multicontinental distributions (Smith et al., 2006; Medina et al., 2011; Patiño and Vanderpoorten, 2018; Krieg and Chambers, 2022). A deeper understanding of the mechanisms of gametophyte dispersal is necessary to assess current biogeographical patterns of plants at multiple taxonomic levels as well as future patterns under climate change, habitat destruction, and other human‐induced changes. Dispersal of gametophytes is also essential in determining plant evolution. For example, in seed plants, pollen dispersal determines the “neighborhood” of randomly mating individuals, with implications for overall population genetic structure, gene flow, inbreeding depression, local adaptation, and speciation (Wright, 1943; Stebbins, 1970; Johnson et al., 2006; Levin and Kerster, 2015). Thus, characterization of pollen dispersal is crucial for understanding the evolution of pollinator systems and of overall population genetic and conservation concerns (Ellstrand, 1992).

The small size of both pollen and spores presents challenges for dispersal (Vogel, 2005a, b; Figures 1 and 2). Both have a low terminal velocity and are rapidly slowed by air, which is advantageous for remaining aloft but means that ballistic propulsion at slow speeds is ineffective (Edwards et al., 2005; Zanatta et al., 2016; Gómez‐Noguez et al., 2017). A solution that has evolved multiple times across the plant tree of life is the use of rapid movements to increase acceleration, velocity, and distance traveled (Martone et al., 2010; Edwards et al., 2019). Many of these rapid movements have been observed or known for centuries, and work in the 19th and first half of the 20th centuries described these fascinating movements. In his books, *Spore Discharge in Land Plants* (Ingold, 1939) and *Spore Liberation* (Ingold, 1965), C. T. Ingold summarized the current understanding of the movements of spores in particular, both in embryophytes and fungi. Ingold describes, for instance, the explosive dispersal of spores in *Sphagnum* L. moss (Sphagnaceae) with detailed illustrations of the varying drying stages of the sporophyte capsule, depictions of cellular components, and in some cases insights from experimental work. These discoveries were made using careful observation of these mechanisms by watching plants in action (under a microscope or not) and with basic microscopic techniques to understand overall cell structure. During the latter half of the 20th century, researchers such as Karl J. Niklas and Josef D. Ackerman devised unique solutions to document rapid movements, such as the use of bubble‐trace photography, particle analysis, and stroboscopic illumination, to define our understanding of abiotic pollination (e.g., Niklas, 1984; Ackerman, 1986, 1997; Niklas et al., 1986).

Although the contributions of these foundational researchers cannot be understated, the speed at which these movements occur makes it difficult to accurately document all stages of mechanical movements in real time or to characterize details such as fluid dynamics or accelerations of small particles. Moreover, differences in biomaterials are not always detectable using standard microscopy techniques. High‐speed video (video filmed at 100–125,000 frames per second [fps]) and analysis of ultrastructure (structure only visible using electron microscopy or other techniques) has enabled further understanding of these extraordinary dispersal mechanisms beyond what researchers were capable of in Ingold's time.

We searched the literature for publications that used high‐speed video and/or microscopy to understand the dispersal of gametophytic diaspores (including spores, gemmae, and pollen; Figures 1 and 2). First, we review the impact and use of high‐speed video in evaluating rapid movements effecting dispersal. We include examples of new discoveries, evaluate the use of different types of high‐speed video cameras, and provide considerations for lighting, staging, and video analysis. Then we link these movements to methods in microscopy techniques, which define the materials and structural elements required for these rapid movements, and discuss how each technique can be used to understand gametophyte dispersal. Finally, we reflect on the importance of understanding gametophyte dispersal for both ecological/evolutionary questions as well as applications in human technologies and future directions.

## HIGH‐SPEED VIDEO

Here we review the impact of high‐speed video on our understanding of gametophyte dispersal and provide an overview of resources for filming rapid motions of plants. The classic spore dispersal studies by Ingold (1939, 1965) relied on careful observation using the tools of the times, but advances in video have carried our understanding of the materials and biomechanics of dispersal to new levels. The visualization of spore and pollen dispersal has been transformed by the development of high‐speed video cameras, some of which are capable of filming at over a million frames per second. Different dispersal mechanisms have evolved independently in several groups from the mosses and liverworts to monilophytes and angiosperms. To understand the evolution of these rapid and propulsive movements, high‐speed video allows the movements to be viewed in slow motion so that the biomechanics can be dissected and the movements can be accurately measured and visualized.

### High‐speed video has revealed new biomechanical mechanisms and features of dispersal systems

We summarize many of the new findings on the biomechanics of rapid movements effecting gametophyte dispersal in Table 1. The contributions of high‐speed video can be categorized into the following: (1) defining new features of plant biomechanics; (2) providing more accurate measures of velocity, acceleration, and terminal velocity; (3) characterizing individual spore behavior; and (4) defining interactions between flowers and their insect visitors. First, high‐speed video has revealed new biomechanical mechanisms of ultrafast motions that were not visible in real‐time video. Notable are the characterization of the stamens of *Cornus canadensis* L. (Cornaceae) as hinged catapults or trebuchets by filming at 10,000 fps (Edwards et al., 2005), the visualization of the spore‐dispersing vortex rings from the exploding capsules of *Sphagnum* moss by filming at 100,000 fps (Whitaker and Edwards, 2010), the three stages of catapulting spores from the leptosporangia of ferns including a built‐in brake system by filming at up to 125,000 fps (Noblin et al., 2012; Poppinga et al., 2015; Llorens et al., 2016), and the dispersal of multiple gemmae from a single droplet hitting a gemmae cup of *Marchantia* by filming at 3000 fps (Edwards et al., 2019).

**Table 1:** Summary of findings revealed through high‐speed video.

Plant species	Findings revealed by high‐speed video	Camera	Lighting	Software and analysis	Film speed (fps)	References
Bryophytes						
*Sphagnum* L. spp. (Sphagnaceae)	*Sphagnum* disperses spores in vortex rings. Measurement of spore launch velocity, acceleration, and movement into the turbulent zone.	RedLake HG‐XL (Integrated Design Tools Inc., Tallahassee, FL, USA)	300‐W Halogen bulbs, fiber‐coupled Xenon discharge lamp, and a 400‐W HMI light powered by a high‐speed ballast	Calculated velocity and acceleration based on successive stills taken from videos	250–100,000	Whitaker and Edwards, 2010
*Brachythecium populeum* (Hedw.) Schimp. (Brachytheciaceae)	Documents the rapid movement of the outer peristome teeth using real‐time video, capturing puffs of spores sifting from a closed capsule using high‐speed video.	MotionPro Y4 (Integrated Design Tools Inc.)	Incident light source (Highlight 3100, Olympus, Tokyo, Japan)	Every seventh image was used to calculate hygroscopic movement of peristome teeth	1000	Gallenmüller et al., 2018
Nine moss species	Measures the variation in settling velocity for spores of nine species of moss, showing the effect of different sized spores and spore ornamentation.	Y4 CMOS (Integrated Design Tools Inc.)	LED backlighting (19 LED Constellation, Integrated Design Tools Inc.) with a 12.5° beam angle on the axis of the camera	Calculated spore settling velocities from five consecutive images	100	Zanatta et al., 2016
Monilophytes						
Seven fern species	Measures the terminal velocity of spores of seven fern species, allowing the behavior (e.g., rotation) to be observed. May allow differences in spore size and ornamentation to be measured.	EXILIM EX‐ZR100 camera (Casio Computer Co., Tokyo, Japan)	Two lights: CREE Q5 LED, 3 W, 800 lumens (Cree Inc., Durham, NC, USA)	Analysis using a Tracker video analysis and modeling tool v. 4.87 (http://physlets.org/tracker/)	1000	Gómez‐Noguez et al., 2017
*Angiopteris evecta* (G. Forst.) Hoffm. (Marrattiaceae)	Documents the sudden cavitation of individual spores, which propels them several millimeters.	Phantom V4.2 B/W Camera (Vision Research, Wayne, NJ, USA)	Lighting not reported	No software reported	2000	Hovenkamp et al., 2009
*Polypodium aureum* L. (Polypodiaceae)	Measures the velocity of spores propelled by the cavitation‐triggered catapult of leptosporangia (10 m/s, 10,000 g). Documents three stages including a built‐in brake (equivalent of a crossbar in medieval catapults). Measures annulus deformation.	Phantom v. 7.11 high‐speed camera (Vision Research)	Camera mounted on microscope (Olympus IX71 or SZX10) (Specific lighting not given)	Custom‐made image analysis routines implemented in MATLAB (MathWorks, Natick, MA, USA)	1–125,000	Noblin et al., 2012; Llorens et al., 2016
*Adiantum peruvianum* Klotzsch (Pteridaceae)	Analysis of leptosporangium motion and tracking ejected spores. Calculation of spore velocity and launch acceleration (6300 g).	MotionPro Y4 (Integrated Design Tools Inc.) with Motion Studio Software	Techno light 270 cold light source (Karl Storz GmbH& Co. KG, Tuttlingen, Germany or Constellation LED's (Integrated Design Tools Inc.) on an Olympus SZX9 dissecting scope or an Axioplan Light Microscope (Zeiss, Oberkochen, Germany)	Motion Studio 1.08.03 (Integrated Design Tools Inc.), and ImageJ (Abramoff et al., 2004; Schneider et al., 2012) and Excel 2007 (Microsoft)	20,000–100,000	Poppinga et al., 2015
*Equisetum arvense* L. (Equisetaceaae)	Documents the “jumps” and “walks” of spores. High‐speed video details the behavior of elaters in response to changes in humidity, which ultimately drive spore movement.	Slow‐motion CCD camera (Marlin, Allied Vision Technology) or H‐S camera Miro 4 (Vision Research)	Lighting not reported	No software reported	7000–120,000	Marmottant et al., 2013
*Equisetum arvense* (Equisetaceaae)	Reports the glide of *Equisetum* spores. Documents that spores falling from the sporangia unfurl their elaters midair, catching the air currents, and switch from a downward to an upward trajectory.	Phantom v310 video camera (Vision Research)	250‐W photo lamps mounted on flexible arms and fiber optic illuminators (Sōlarc model: LB50; Ushio America, Cypress, CA, USA)	No software reported	1000	Edwards et al., 2019
Angiosperms						
*Cornus canadensis* L. (Cornaceae)	Demonstrates the trebuchet mechanism of stamens. Provides measurements of spore velocity and acceleration.	Motion Xtra HG‐100K (DEL Imaging Systems, Cheshire, CT, USA)	Xenon discharge lamp and two 250‐W incandescent lamps	Individual frames were imported into Adobe Photoshop (Adobe, San Jose, CA, USA) and distances of movement measured. Successive vertical frames were used to measure terminal velocity of pollen	1000–10,000	Edwards et al., 2005
*Morus alba* L. (Moraceae)	Shows explosive propulsion of pollen in the white mulberry. Documents the catapult system where the stamen filament straightens in less than 25 ms.	Photron Fastcam (Photron USA, San Diego, CA, USA)	Fiber‐lite High Intensity Illuminator, Series 180 (Dolan‐Jenner Industries, Inc., Boxborough, MA, USA)	Custom software written in MATLAB (MathWorks)	120,000	Taylor et al., 2006
*Kalmia latifolia* L. (Ericaceae)	Documents insect behavior on flowers, showing bees "pulling" stamens out of pockets. Measures pollen dispersal (trajectories and ranges).	FASTCAM SA3 with a 105 mmf/2.8 lens (Photron USA)	Natural lighting in an arboretum	Digitized videos using the MATLAB‐based program DLTdv5 (Hendrick, 2008; MATLAB R2014b, MathWorks)	5000	Switzer et al., 2018
*Hydrilla verticillata* (L. f.) Royle (Hydrocharitaceae)	Visualizes the unfolding of stamens and explosive propulsion of pollen.	FASTCAM SA3 with a 35 mm f/2.0 lens (Photron USA)	Lighting not reported	MATLAB‐based program DLTdv5 (Hendrick, 2008)	5000	Zhang et al., 2020
*Plantago lanceolata* L. (Plantaginaceae)	Visualizes the turbulence‐induced resonance vibration that facilitates pollen release.	Casio Elixim FH25 digital camera (Casio Computer Co.)	Lighting not reported	Tracker Video and Analysis and Modeling Software (Open Source Physics, http://www.compadre.org/osp/). OriginPro v. 8.6.0 Sr2 (OriginLab, Northampton, MA, USA)	120	Timerman et al., 2014
*Thalictrum* L. spp. (Ranunculaceae)	Documents vibrational response of stamens to air flow patterns for 36 spp. of *Thalictrum*	Casio Elixim FH25 (Casio Computer Co.) and Sony a6300 (Sony Corporation, Tokyo, Japan) digital cameras	Lighting not reported	Open‐source Tracker Video Analysis and Modeling Tool (https://physlets.org/tracker/)	120	Timerman and Barrett, 2018, 2019
*Catasetum fimbriatum* Lindl. & Paxton (Orchidaceae)	Computes the velocity, speed, acceleration force, and kinetic energy of hair‐triggered released pollinia.	Phantom V5.0 high‐speed digital imaging system (Vision Research)	A single halogen lamp	Phantom CineViewer 606 (Vision Research) and Logger Pro 3 (Vernier, Beaverton, OR, USA)	1000	Nicholson et al., 2008
*Hypochaeris radicata* L. (Asteraceae)	Reports the styles act like miniature catapults when pushed by visiting bees.	Olympus high‐speed camera	Lighting not reported	Open‐source Tracker Video Analysis and Modeling Tool (https://physlets.org/tracker/) and custom scripts in MATLAB (MathWorks)	5000	Ito et al., 2020
*Cyclamen persicum* Mill. (Primulaceae), *Exacum affine* Balf. f. (Gentianaceae), *Solanum dulcamara* L., and *S. houstonii* Martyn (Solanaceae)	Compares the vibrations in stamens stimulated by the thoracic vibrations of bees for four species with different stamen architecture, suggesting the co‐evolution between bee behavior and stamen architecture.	FASTCAM SA‐8 camera (Photron USA)	Halogen bulbs	DLTdv7 digitizing tool in MATLAB 9. DLTdv5 (Hedrick, 2008); MATLAB R2014b (MathWorks)	6000	Nevard et al., 2021
*Clerodendron trichotomum* Thunb. (Lamiaceae)	Uses high‐speed video to slow down insect behavior; shows visits of four different insect visitors differ with respect to contacts with anthers/stigmas and nectar drinking times.	High Speed EXILIM EX‐F1 (Casio Computer Co.)	Natural lighting in the field	No additional software	Film speed not reported	Sakamoto et al., 2012
*Solanum lycopersicum* L. (Solanaceae)	Shows that bumble bees and blue‐banded bees handle buzz release of pollen from poricidal anthers differently.	TS3 (Fastec Imaging, San Diego, CA, USA)	Natural lighting in a botanical garden	No additional software	2000	Switzer et al., 2016

*Note*: fps = frames per second.

Second, high‐speed videos of falling spores have provided more precise measures of terminal velocity, acceleration, or falling rates of spores, which when compared, may provide insight on the evolution of the diversity of spore and pollen sizes and impact of ornamentation patterns. Some examples of the diversity in size, shape, and ornamentation of gametophyte diaspores are given in Figures 1 and 2. Zanatta et al. (2016) and Gómez‐Noguez et al. (2017) link differences in size to terminal velocities for ferns and mosses. Examples of acceleration measurements include the catapult stamens of male flowers of *Morus alba* L. (Moraceae), which straighten in 25 µs (Taylor et al., 2006), or the astounding acceleration achieved by the stamens of *Cornus canadensis*, which accelerate at up to 24,000 m·s^−2^ (Edwards et al., 2005; Video 1).

**Video 1:** Video of an exploding *Cornus canadensis* flower filmed at 10,000 fps shows that the stamens are miniature hinged catapults (trebuchets) flinging pollen upwards where it can affix to a visiting insect or be carried by the wind. File is 292 KB in size, 350p, for a real‐time length of approximately 12 milliseconds. Playback is at 15 fps. Video credit: A. Acosta, J. Edwards, M. Laskowski, and D. Whitaker. To view this version, please visit https://onlinelibrary.wiley.com/doi/10.1002/aps3.11463.

Third, individual spore behavior effecting dispersal has been discovered through high‐speed video. Spores of the fern *Angiopteris evecta* (G. Forst.) Hoffm. (Marattiaceae) filmed at 2000 fps show that a sudden cavitation of the spore propels it several millimeters (Hovenkamp et al., 2009). A relatively slow frame rate of 1000 fps captures the mid‐fall unfolding of the elaters on the individual spores of the horsetail, *Equisetum* L. (Equisetaceae), which reverse their trajectory from downward to upward, documenting the importance of elaters in facilitating the glide of these spores (Edwards et al., 2019; Figure 1C).

Finally, high‐speed video can also add to our understanding of flower–pollinator interactions. With an estimated 87.5% of angiosperms pollinated by animals (Ollerton et al., 2019), flower–pollinator interactions are critical for understanding pollen dispersal. Insect handling of flowers can occur too quickly to be captured by the naked eye or traditional video, which records at 30 fps (Sakamoto et al., 2012; Switzer et al., 2016). Slow‐motion video can detail the behavior of pollinators during flower visitation so that one can see if visitors are collecting pollen, eating pollen, and how they are manipulating the flower to gather resources. Ultimately slow‐motion video allows the observer to score whether pollen is being properly placed for dispersal to conspecific plants. Sakamoto et al. (2012) used high‐speed video to observe fine‐scale flower‐visiting behavior by three different insects to *Clerodendrum trichotomum* Thunb. (Lamiaceae). They show pollinator efficiency varies, with the hawkmoth (*Macroglossum pyrrhosticta*) making fewer contacts with anthers and/or stigma than those made by the butterfly (*Papilio dehaanii*) or carpenter bee (*Xylocopa appendiculata*). Switzer et al. (2018) filmed flower visitors to *Kalmia latifolia* L. (Ericaceae) at 5000 fps and were able to document bumble bees, the main pollinators, actively pulling stamens out of the petal pockets. Below we report on the use of a high‐speed video camera and an iPhone camera to film insect visitors to flowers, which can define how the insects interact with the flowers they visit.

### Considerations for filming high‐speed video

Knowing the natural history and specifics of the study plant related to dispersal (such as phenology or morphological changes indicative of dispersal) is important for getting useful video footage and interpreting the adaptive significance of the fast motion. Although most filming to date has been done in the lab, knowledge of a plant's habitat and behavior is critical to getting the best material and interpreting the plant's responses. For example, in western Massachusetts and southern Vermont, *Sphagnum* moss sporulates in mid to late July. It grows in bogs and disperses its spores on warm sunny days. Although drying is the principal driving force for capsule explosion, the capsule must be mature to explode. We observed that mature capsules typically cavitate on one side, an indication that it will explode within approximately 15 min (personal observations; Whitaker and Edwards, 2010). Similarly for the flowers of bunchberry dogwood, we could tell a flower was ready to explode when the stamen filaments were bent and fully protruded from between the petals, forming an “X” when viewed from above. Ingold (1939) mentions a similar maturation requirement for the capsules of the leafy liverwort *Frullania dilatata* (L.) Dumort. (Frullaniaceae), where dehiscence can only be induced by drying if the capsules are “perfectly ripe.”

Equally important is the staging for filming. Features to consider include physical setup, frame rate, lighting, air movement, electrostatic interference, direction of filming, magnification, and film quality. It is useful to look at the successful setups used for the studies reported in Table 1. In general, frame rate needs to slow the motion sufficiently to dissect the biomechanics of the movement, and thus will vary depending upon the speed of the movement. If the frame rate is too slow, the movement may be blurred, while filming at a speed much faster than needed can lead to unnecessary financial costs and difficulties in processing the video. For example, at 1000 fps the floral explosion of bunchberry dogwood is blurred (Appendix S1), whereas at 10,000 fps the motions of the stamens and petals can be distinguished despite some blurring (Video 1).

Because the structures filmed for dispersal in gametophytes are often small, camera setups for high‐speed filming that allow making small adjustments increase the probability of getting good footage. The camera should be placed on a sturdy tripod that has an easily adjustable up‐and‐down crank and maintains its position once set. Staging equipment that allows micromanipulations in the x‐, y‐, and z‐planes is critical to provide the sharpest focus and framing of the subject. For example, our setup for filming the splash cups of *Marchantia* included filming through a dissecting microscope on an adjustable platform, special considerations for exposure to water droplets, protection from air currents, and backlighting specific to this system (Appendix S2).

Lighting used in high‐speed filming varies both in the number of lights and types of lighting (Table 1). Lighting can be used effectively from the top, side, or even back of the subject if it is transparent and illumination through the plant can be useful. New cameras can film at lower light intensities, which increases the options for lighting, including lights that are reasonably priced. LED lights used in several studies (Poppinga et al., 2015; Zanatta et al., 2016; Gómez‐Noguez et al., 2017) have the advantage of providing high light intensity yet remaining cool. Multiple sources of light help to eliminate shadows. Any lights powered by AC current (60 Hz in the United States, 50 or 60 Hz depending on location outside of the United States) can potentially cause a flicker if the filming speed and line frequency are at odds. Possible solutions to a flicker include adjusting the film speed, using multiple light sources, using lights powered by a low‐ripple DC source, or using sunlight, which is constant.

Air currents and electrostatic interference with spore movement also require careful consideration. For example, Zanatta et al. (2016), when measuring fall rates of moss spores, used antistatic‐treated clear PVC tubes both to minimize air movement and to reduce electrostatic interference. Gómez‐Noguez et al. (2017), when measuring the terminal velocity of fern spores, used a glass tube covered with aluminum foil, which functioned like a Faraday cage, and then grounded the entire system to physical earth. For studying droplet dispersal of gemmae from *Marchantia* gemmae cups, we used a clear acrylic tube to minimize air flow and increase the number of drops hitting the cups (Appendix S2; Edwards et al., 2019).

The direction of filming and magnification are critical. For measurements taken from successive frames, filming perpendicular to the direction of movement is important so that the full range of motion can be analyzed. Magnification is also important as images taken at higher magnifications are useful for dissecting the biomechanics of an action (e.g., the vortex ring of *Sphagnum* moss or the trebuchet of *Cornus canadensis* flowers), whereas distant shots can reveal patterns of spore dispersal (e.g., the height of a *Sphagnum* spore cloud).

Analysis of videos often involves measurements taken from successive still frames of the video, which allows the measurement of distances moved during known time intervals. Based on these data, velocities and accelerations can be calculated. Programs that are helpful in these measurements and calculations include the open‐source imaging software ImageJ (Abramoff et al., 2004; Schneider et al., 2012), Adobe Photoshop (Adobe, San Jose, California, USA), and MATLAB (MathWorks, Natick, Massachusetts, USA), which has a digitizing program (DLTdv5; Hedrick, 2008) and can also be used to fashion custom software (Table 1; Taylor et al., 2006; Ito et al., 2020). Several studies also report using a tracker video analysis software (Gómez‐Noguez et al., 2017; Timerman and Barrett, 2018, 2019; Ito et al., 2020).

Finally, computational power and storage can become issues when dealing with many or large video files, particularly if you need to save individual frames for analysis. In our studies, we use 1 or 2 TB external hard drives for storing videos. For some high‐speed video systems, software compatibility is important. The software for our video system only worked with the Microsoft Windows (Microsoft, Redmond, Washington, USA) format. However, once videos and images were produced, analysis could be done on either Windows or Macintosh machines. Our analyses were mostly done on MacBook Pros (Apple Inc., Cupertino, California, USA) without any supplementary computational power. We note that technologies for computational storage and power are continually changing, and that there are many systems that are suitable for storing and working with these types of data.

### Review of high‐speed video cameras

**Figure 3:**
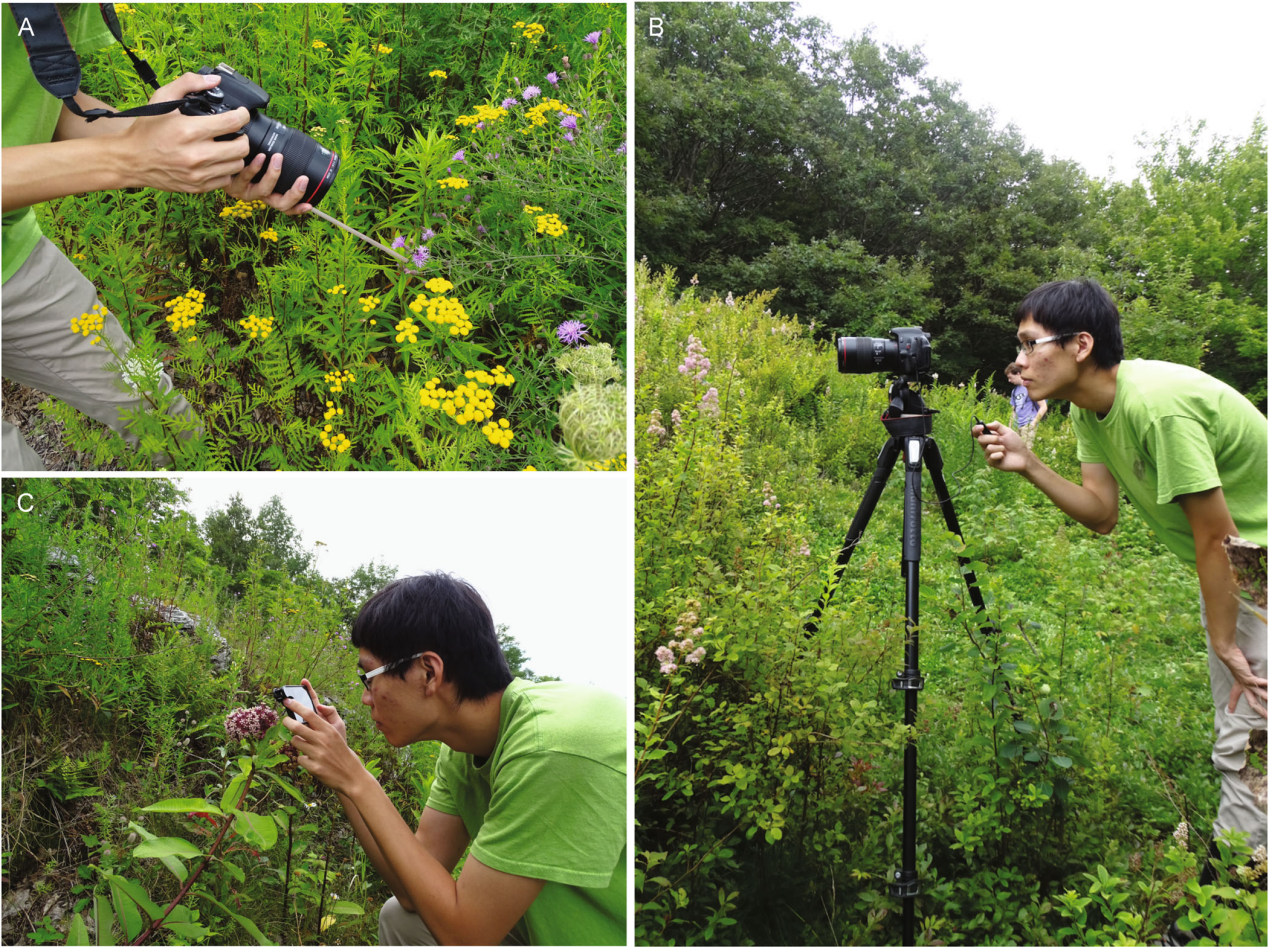
Examples of using high‐speed video cameras for filming pollinators in situ by D. D. Wang. (A) Use of hand‐held point‐and‐shoot camera with post‐event triggering and a straw to indicate focal distance. (B) Point‐and‐shoot camera on tripod with zoomed lens, cable release, and a post‐event trigger system. (C) iPhone hand‐held camera with Anazalea macro lens attached.

Video cameras have continued to improve and become more accessible, such that filming with the most sophisticated professional high‐speed cameras can achieve frame rates as high as over one million frames per second. Although these professional models are expensive, the development of affordable options has made high‐speed video more broadly accessible. We group high‐speed videos into three categories: high‐end professional cameras, point‐and‐shoot cameras, and iPhone (Apple) cameras (Figure 3).

Much of the published work uses professional cameras (see the list of models used in Table 1), and these professional high‐speed cameras have the benefit of the greatest range of frame rates, custom options for dpi (dots per inch) and shape of the filming area, and, critically, post‐event triggering, where the saved frames are constantly buffered so that once the camera is triggered, the last frames filmed are saved. These professional cameras are used most successfully in a lab setting where staging elements can be controlled.

Both the compact point‐and‐shoot cameras and iPhones are portable and are most useful for filming in the field where plants can be observed in situ. The small size of these video systems makes them easier to use in the field than the larger professional high‐speed cameras, and their lower frame rates are sufficient for many studies, including observations of pollinator behavior in the field. Both are easily hand‐held to capture short events, and the point‐and‐shoot camera has a remote trigger option, which is useful when filming using a tripod setup. The point‐and‐shoot cameras can film up to 960 fps, while all iPhone models with “SLO‐MO” (slow motion) capability will record videos at 120 fps, and newer models (iPhone 8 and newer) that are powered by the A11 Bionic chip can record at up to 240 fps. We have used these cameras successfully to capture the behavior of insects visiting flowers and discuss the merits of each below.

We used a point‐and‐shoot camera with high‐speed capabilities (HFR mode on a Sony DCS‐RX10M4 [Sony Corporation, Tokyo, Japan]) to film visitors to flowers. This camera has the option of filming at 240, 480, or 960 fps. It also has the option of post‐event triggering, where the camera is constantly buffering and, when triggered, will save the last frames filmed. We used this camera in three setups: hand‐held, hand‐held with a straw to indicate focal distance (Figure 3A), and on a tripod with a cable release (see Figure 3B, Videos S1–S3). The advantages of this system are the higher filming rates (up to 960 fps); post‐event triggering; playback speeds of 24, 30, or 60 fps; and tripod setup with cable release, which allows greater distance from the subject. The drawbacks are that the focal distance and exposure are set once filming has started, there is limited film time (e.g., at the fastest rate of 960 fps only 3 s can be filmed), and the video quality decreases with increasing frames per second. As an example of this last drawback, see the series of three videos of insects visiting *Spiraea alba* Du Roi (Rosaceae) filmed at 240, 480, and 960 fps (Videos S1–S3). These video files are 182, 198, and 348 MB in size (1080p), for real‐time lengths of approximately 3.2, 1.6, and 1.4 seconds, respectively. A final drawback is that, when saving videos, the camera is not available for use during the next shoot (e.g., saving can take up to 2 min 38 s when saving a 960 fps video filmed with quality priority).

We have used the iPhone 12 Mini (Apple Inc.) with the ProMovie Version 1.10 app (Panda Apps Limited, Cumbernauld, United Kingdom) and an added macro lens (Anazalea 15X HD macro lens, model A‐FL‐BW; Schott Optical Glass, Mainz, Germany) to film insects visiting flowers (Figure 3C). The ProMovie app allows for the manual adjustment of the shutter speed, ISO, white balance, and focal distance. The macro lens allows close filming, but distant filming without the added lens is also useful. We used this video system to capture slow‐motion videos of insect behavior on flowers. In Video 2, we show that when the hoverfly (*Syrphus* sp.) visits the flowers of *Diervilla lonicera* Mill., it collects pollen, feeding first from the anther sac and then feeding directly off the stigma. This file is 60 MB in size, 1080p, for a real‐time length of 6.25 s. The advantages of the iPhone setup include portability, affordability, high‐resolution video (1080p), video length that typically is not limited, macro lens options, and automatic adjustable focusing and exposure while filming. The drawbacks are that the maximum film speed is only up to 240 fps and the focal distance with the macro lens is short (5 cm), making it difficult to film insects that are easily scared off. For filming without the macro lens, the shortest filming distance is 7 cm. The playback speed for iPhone videos is set at 30 fps but can be changed after recording using video editing software.

**Video 2:** Syrphid fly (*Syrphus* sp.) eating pollen directly from the anther sac and from the stigma of *Diervilla lonicera* demonstrates pollinivory and shows how the tongue of the fly works. Video filmed at 240 fps using a hand‐held iPhone 12 mini with an attached Anazalea macro lens. File is 60 MB in size, 1080p, for a real‐time length of 6.25 s. Playback is at 30 fps. Video credit: D. Wang. To view this version, please visit https://onlinelibrary.wiley.com/doi/10.1002/aps3.11463.

Ultimately, the choice of a camera system for high‐speed video depends upon the study question, details of the study system, as well as cost and filming location. Although we have summarized some advantages and disadvantages of a subset of example cameras above, there are many options for each broad camera category (as exemplified by the options for professional cameras described in Table 1). Still, these low‐cost options are excellent for filming pollinators in the field, are a good starting point for documenting high‐speed plant motions, and can help to provide preliminary evidence or data to potentially support more expensive cameras if necessary.

## MICROSCOPY AND ULTRASTRUCTURE

### Microscopy reveals structure at multiple levels

Our understanding of gametophyte dispersal has been further enhanced by advanced microscopy techniques at different levels of organization. As early as the 19th century, botanists described some of the most spectacular spore dispersal mechanisms without the use of high‐speed video or modern microscopy. For example, Nawaschin (1897) detailed the explosion of *Sphagnum* moss capsules and both Goebel (1895) and Kamerling (1898) provided early descriptions of the explosive dehiscence of the sporangia of the liverwort *Frullania* Raddi. In both *Spore Discharge in Land Plants* and *Spore Liberation*, Ingold (1939, 1965) summarized these early works and added his own studies detailing the morphological and cellular structures of the organs and appendages involved in spore dispersal, including extensive explanations of these structures as well as illustrations. Observation using both dissecting and compound microscopes has enabled researchers to understand the overall morphological structures (e.g., the peristome, annulus, capsule shapes, presence of elaters), as well as the anatomical features (e.g., cell types and shapes, differential cell wall thicknesses) and other histological features used in the dispersal of spores (Ingold, 1939, 1965). More recent work has expanded on these observations and basic microscopy methods by enabling the examination of more detailed ultrastructure using different types of electron microscopy as well as advanced techniques, such as X‐ray computed tomography or fluorescence microscopy. The combination of diverse microscopy techniques, along with high‐speed video, allows for an understanding of the structure and biomaterials involved in gametophyte dispersal at multiple levels of organismal organization. Here we highlight the diversity and utility of major microscopic techniques, some special considerations for each, and new insights into gametophyte dispersal elucidated by each technique (Table 2).

**Table 2:** Major microscopy methods, their utility, approximate magnification, special considerations, and examples of their utility in understanding gametophyte dispersal.

Microscopy method	Utility	Magnification range	Special considerations	Examples
Dissecting/stereomicroscopy	External morphology	1–100×	Focal plane	Whole inflorescence structure (Taylor et al., 2006; Edwards et al., 2019; Zhang et al., 2020)
Compound/light microscopy	Cell types and shapes, staining for compounds, sectioning through materials	40–1000×	Fixation, staining, etc.	Capsule walls and dehiscence grooves (Duckett and Pressel, 2019), arrangement of organs (Taylor et al., 2006; Whitaker et al., 2007; Zhang et al., 2020), water‐absorbing potential (Taylor et al., 2006; Zhang et al., 2020), cavitation and gas bubbles (Hovenkamp et al., 2009; Poppinga et al., 2015)
SEM	Ultrastructure of surfaces (spores, dispersal organs, etc.)	~1,000,000×	Standard or cryopreservation, environmental conditions	Characteristics of diaspores (Pohjamo et al., 2006; Hovenkamp et al., 2009; Poppinga et al., 2015; Zanatta et al., 2016; Gómez‐Noguez et al., 2017), sporophyte dispersal organs (Hovenkamp et al., 2009; Poppinga et al., 2015; Duckett and Pressel, 2019; Zhang et al., 2020), mechanism (Hovenkamp et al., 2009; Poppinga et al., 2015; Duckett and Pressel, 2019), elemental analysis (Volkov et al., 2019)
TEM	Subcellular structures	~2,000,000×	Where to section	Distinctive cytology in different areas (Hovenkamp et al., 2009; Duckett and Pressel, 2019)

*Note*: SEM = scanning electron microscopy; TEM = transmission electron microscopy.

### Dissecting and compound light microscopy

Dissecting microscope (stereo microscope) and light (compound) microscope images are still invaluable for observing the overall external morphological structure and organization of the organs involved in the dispersal of gametophytes. Magnifications of up to 100× are crucial for observing relatively small structures such as anthers involved in pollen dispersal, and specimens can be observed at varying stages of dehiscence (pre‐ versus post‐dispersal). Images captured using dissecting microscopes have been used to observe the mechanics of explosive pollination in flowering plants, including the positioning and shape of floral organs that allow for the “catapult”‐like dehiscence of stamens documented by high‐speed video (Taylor et al., 2006; Edwards et al., 2019; Zhang et al., 2020; Table 2). For instance, in the aquatic flowering plant *Hydrilla verticillata* (L. f.) Royle (Hydrocharitaceae), dissecting microscope images of male flowers before, during, and after anther movement displayed the positioning and importance of the sepals and the overall catapult‐like structure involved in pollen dispersal (Zhang et al., 2020). Dissecting microscopes have a limited field of range, but specialized hardware and software, such as the focus stacking system of Macroscopic Solutions (Macroscopic Solutions LLC, Tolland, Connecticut, USA) or Helicon Focus (Helicon Soft Ltd., Kharkiv, Ukraine) can take multiple images of samples on multiple focal planes and stack them into clear and accurate images for samples >10 µm (Brecko et al., 2014; Cameron and Zaspel, 2019).

Fixation of materials, sectioning, and staining for light microscopy allow for the observation of organs and cells layer by layer throughout the three‐dimensional structure of the organ(s) of interest at up to 1000× magnification. Viewing longitudinal sections of organs in enclosed buds and floral structures helps to understand the development of these organs and how their positioning affects explosive pollination, beyond what is observable on the surface through high‐speed video or when viewed using a dissecting microscope (Taylor et al., 2006; Whitaker et al., 2007; Zhang et al., 2020). Furthermore, staining of these organs can also lead to insights into chemical composition. For instance, in the explosive flowers of *Cornus canadensis* (described above), staining of organs with ruthenium red and hydroxylamine hydrochloride–ferric chloride revealed that cell walls in the “hinge” section of stamens are high in acidic pectin and low in esterified pectin, resulting in tissue softening (Edwards et al., 2019). Light microscope images are also used in the identification of the shapes and water‐absorbing abilities of some cells involved in these rapid movements (Taylor et al., 2006; Zhang et al., 2020). In non‐flowering plants, light microscopy has allowed for more detailed observation of the dehiscence of moss and liverwort sporophyte capsules (Gallenmüller et al., 2018; Duckett and Pressel, 2019) and has elucidated the importance of gas bubbles and cavitation in the dispersal of fern spores (Hovenkamp et al., 2009; Poppinga et al., 2015). Basic histology and light microscopy are still essential for understanding gametophyte dispersal and for highlighting regions needing further investigation of ultrastructure.

### Electron microscopy

Although transmission electron microscopy (TEM) and scanning electron microscopy (SEM) were in use during Ingold's time, they were not yet commonly used to understand gametophyte dispersal. Using SEM, researchers can observe surface topography at over 1,000,000× magnification while imaging a greater field of depth than a light microscope. SEM can also image the surface of objects without destroying the sample (Vernon‐Parry, 2000). The standard method of SEM, as well as cryo‐SEM (SEM conducted under very low temperatures) and environmental SEM (conducted in gaseous environments, reviewed in Danilatos, 1991), has furthered the understanding of ultrastructure in spores and the organs involved in gametophyte dispersal (Table 2). SEM has been particularly useful in examining and characterizing the shape, size, and ornamentation of individual spores in mosses and ferns, factors that often affect dispersal ability through the air (Figure 1; Hovenkamp et al., 2009; Poppinga et al., 2015; Zanatta et al., 2016; Gómez‐Noguez et al., 2017). SEM has also been used to observe sporophyte organs responsible for spore and pollen dispersal, including capsules, anthers, and sporangia (Hovenkamp et al., 2009; Poppinga et al., 2015; Duckett and Pressel, 2019; Zhang et al., 2020). We used SEM to visualize the external structure, shape, and size of gametophytic diaspores, including spores, gemmae, elaters, and pollen (both as individual grains or packaged together in pollinia or viscin threads) (Figures 1 and 2; Edwards et al., 2019). Furthermore, comparison of SEM images taken before versus after dispersal, in whole versus ruptured organs, and in various states of hydration can lead to further insights into mechanism as well as structure (Pohjamo et al., 2006; Hovenkamp et al., 2009; Poppinga et al., 2015; Duckett and Pressel, 2019).

TEM is used to image specimens at the near atomic level (approximately 2,000,000× magnification) and, thus, for our purposes, can be used to image subcellular components in plants (Table 2). Unlike SEM, TEM must be performed on thin sections of a sample and is performed destructively. TEM is likely most well known in plants for the imaging of different cells, cell types, and foundational work in phloem tissues (Esau and Cheadle, 1961). Studies using TEM typically build on discoveries made using other techniques. For example, in their study of capsules and elaters of the liverwort *Haplomitrium* Nees (Haplomitriaceae), Duckett and Pressel (2019) used TEM to detail the cytology of cells of the capsule walls in terms of the vacuoles, plastids, and endoplasmic reticulum, and notably found a lack of plasmodesmata in capsule walls that split (but the presence of plasmodesmata in those that did not break open). The use of TEM also allowed Duckett and Pressel (2019) to disprove the previous idea that there was a breakdown of cellular contents in the mature cells of the capsule wall. Similarly, Hovenkamp et al. (2009) used TEM to observe differences in the spore wall layers in the fern *Angiopteris evecta*. Paired with high‐speed video documenting cavitation of the spore, TEM helped the authors to conclude that action of the spore walls (not the sporangium) drives spore ejection in this system. We suggest that future studies use TEM to study the subcellular components that differ between cell layers or tissues in the organs of plants involved in explosive gametophyte dispersal.

### Additional microscopy techniques and considerations

Some studies have moved beyond the above‐mentioned microscopy techniques to gain additional insights into gametophyte dispersal. The combination of SEM with chemical spectral techniques displays the distribution of elements and polymers involved in spore dispersal in the horsetail *Equisetum* (Volkov et al., 2019). 3D images can be obtained using X‐ray computed tomography and used to further understand soft tissue structure (Staedler et al., 2013). Dellinger et al. (2014) used this technique (along with SEM and light microscopy) to study puff pollination in the bird‐pollinated flowering plant genus *Axinaea* Ruiz & Pav. (Melastomataceae). They documented the presence and location of vasculature, pollen chambers, and aerenchyma in the stamens involved in the “bellows” action that propels pollen in this system.

Finally, beyond the type of microscopy used, the preparation of materials is also important (similar to the staging of materials for high‐speed video). Ingold recognized the importance of hydration status in many of these dispersal mechanisms, and recent studies have used different preparation techniques to observe tissues with different hydration statuses and under different environmental conditions during the actual microscopic imaging process. For light microscopy, comparisons of spore samples observed in either water, lactic acid (to inhibit rehydration), or glycerol (to induce dehydration) confirmed the role of cavitation in creating gas bubbles that facilitate spore dispersal in the fern *Angiopteris evecta*; this was also documented visually using high‐speed video and TEM (Hovenkamp et al., 2009). The use of cryo‐SEM allows for samples to be flash‐frozen in either hydrated or partly dehydrated states and then imaged at low temperatures, preventing changes in hydration status during processing (Duckett and Pressel, 2019). While the high vacuum of standard SEM requires that samples be dried during processing, environmental SEM can be used on fully hydrated samples, thus offering another way to observe and compare specimens in different states of hydration (Danilatos, 1991). An example of the use of environmental SEM is the study by Katifori et al. (2010) reporting how pollen grains respond to changes in hydration, folding up by curling inward when dry and expanding after landing on a wet stigmatic surface. As another example, we used environmental SEM to image hydrated samples of *Marchantia* gemmae, which rapidly lose water and change shape when drying. This use of environmental SEM allowed us to determine the actual size and shape of the hydrated gemmae (Figure 1B). These preparations and conditions thus enable researchers to investigate changes in ultrastructure that are most likely of utmost importance in gametophyte dispersal.

## FUTURE DIRECTIONS

In this review, we summarized how the combination of high‐speed video techniques and microscopy of ultrastructure of plant materials has enabled the discovery of novel mechanisms of gametophyte dispersal across the plant tree of life. We emphasize that the nature of these rapid movements involving the projection of small diaspores can be understood more fully with the ability to film at very high frame rates that can then be slowed down, while simultaneously visualizing the organ‐level, tissue‐level, cellular, and/or subcellular components involved using advanced microscopy. Here, we discuss future directions for the use of high‐speed video and microscopy in understanding rapid movements in plants, some limitations of these methods, and finally how insights from these movements can inform human technologies through biomimetics.

### Additional dispersal mechanisms to assess

Even given the breadth of studies reviewed here, we believe there are additional avenues for advancement in terms of high‐speed video, microscopy, and gametophyte dispersal. Dispersal systems described prior to the development of high‐speed video, or described later but not including slow‐motion photography, might be better understood when filmed with high‐speed video. Notably, some of the detailed studies of bryophytes reported by 19th‐century botanists (e.g., Nawaschin, 1897; Kamerling, 1898) and then by Ingold (1939, 1965) have not, to our knowledge, been filmed at high speeds. This includes the exploding sporophytes of leafy liverworts exemplified by *Frullania dilatata* (Kamerling, 1898; Ingold, 1939) and *Cephalozia bicuspidata* (L.) Dumort. (Cephaloziaceae; Ingold, 1965; Laaka‐Lindberg and Syrjänen, 2013), where spiral elaters are affixed by one end to the internal wall of the liverwort capsule and act as miniature springs, flinging the spores away when the capsule splits open when mature and dried. Angiosperm systems are too numerous to cover extensively here, but as an example, the biomechanics of the many species of flowers in the subfamily Faboideae (Fabaceae), for which exposure to the style and pollen release depend on the keel being depressed (e.g., alfalfa flowers), could be better defined by high‐speed video.

Beyond biomechanics of the moving parts, high‐speed video can also be used to assess settling velocities in pollen or spores that have different shapes, sizes, or other features (Niklas, 1985). Stroboscopic photography has been used in this context (e.g., determining settling velocities in *Ephedra* L. pollen; Bolinder et al., 2015) as has high‐speed video in some cases (Gómez‐Noguez et al., 2017), but many taxonomic groups remain underexplored. The spread of haploid propagules in aquatic environments is another relatively understudied avenue in research, especially in terms of high‐speed video. For example, submerged mosses such as *Fontinalis antipyretica* Hedw. can produce numerous types of asexual haploid propagules in addition to rare spore production (Ares et al., 2014), but little is known about how far they disperse in the water or their overall movement underwater. Filming in tanks in a laboratory setting (as done for ballistic pollination of *Hydrilla verticillata* by Zhang et al., 2020) under different flow rates could assess how far these different propagules travel, related both to environmental conditions and the shape or size of the propagules. Traditional video has been used in this type of setting to examine coral larvae settling speeds and capabilities (Hata et al., 2017), and we think that high‐speed video could be of additional use in a number of underwater systems.

Fortunately, high‐speed video is becoming more accessible (Table 1), thereby decreasing barriers to entry and providing additional opportunities for natural history observations to lead to more detailed, technical studies. In particular, the use of more portable high‐speed cameras (such as the iPhone) will enable additional filming in the field, where gametophyte dispersal occurs under more realistic abiotic conditions and with biotic partners. Successful filming in situ has documented abiotic pollination in natural conditions (Timerman et al., 2014; Timerman and Barrett, 2021), as well as detailed interactions between flowers and biotic pollinators (Sakamoto et al., 2012; Switzer et al., 2016; Ito et al., 2020). Here we have included additional video examples of pollinators visiting flowers in situ (Videos 2, S1–S3), which has, in part, been facilitated by the enhanced ability to film under ambient light conditions and using more portable systems. An increased breadth of understanding of these mechanisms can then be followed up by assessing the physiological mechanisms and composite structures that enable these movements.

There are also many rapid plant movements that do not involve gametophyte dispersal that have been documented using the methods described above. We briefly mention these examples, as the focus of this review is on gametophyte dispersal. For instance, many species across the angiosperm phylogeny employ ballistic seed dispersal, which also involves rapid movements, and can be documented with high‐speed video. Video can be used to document the movements and calculate energy transfer and ejection velocities, as has been done in *Impatiens capensis* Meerb., *Hamamelis* L., and *Oxalis* L. species, as just a few examples (Hayashi et al., 2009; Edwards et al., 2019; Poppinga et al., 2019; Li et al., 2020). Other plant motions related to nutrition have been documented, such as in the carnivorous plants *Aldrovanda* L. or *Utricularia* L. (Vincent et al., 2011; Westermeier et al., 2018), and additional mechanisms can be investigated in terms of their biomechanics and physics (Skotheim, 2005).

Analysis of ultrastructure can add additional insights into the above‐mentioned mechanisms, as well as highlighting additional structural and chemical aspects of the involved tissues. Developmental plant biologists have been at the cutting edge of bioimaging, especially using live imaging techniques, many of which could be applied to document the development and structure of dispersal organs and propagules (reviewed in Sappl and Heisler, 2013; Prunet and Duncan, 2020). For instance, the use of cryo‐SEM and environmental SEM to investigate ultrastructure under various environmental conditions can facilitate the characterization of diaspores in more realistic settings and complement high‐speed video.

High‐resolution X‐ray computed tomography can lead to exciting new insights into gametophyte dispersal, such as additional documentation of the three‐dimensional soft tissue structures involved, or the speed and direction of water movement that drives many high‐speed motions. X‐ray micro‐imaging has also been used to document embolism repair in xylem in vivo (Brodersen et al., 2010), and filming with higher‐speed charge‐coupled device cameras could potentially document the role of water in driving these (and other) biomechanical dispersal mechanisms. Other novel microscopic techniques, such as confocal microscopy, can take advantage of autofluorescence in plants for anatomical observation and are applicable across green plants (Pegg et al., 2021), contributing to a phylogenetic perspective on the evolution of dispersal.

Here, we have focused on the current and potential uses for high‐speed video in understanding rapid plant movements. We do acknowledge that current technologies cannot document all potential plant movements. For instance, present‐day cameras are limited by depth of field, which can present difficulties in tracking movements of microscopic materials. In some cases, an understanding of the natural history and triggers for dispersal is lacking, which makes filming inaccessible. For example, little is known about the sporophyte generation and spore dispersal mechanisms in deep‐water mosses (Ares et al., 2014).

### Inspiration for human technologies

We have made comparisons between some of these botanical dispersal mechanisms and human‐built structures, such as the “trebuchets” of *Cornus canadensis*, which serve as useful comparisons for visualization and science communication (Edwards et al., 2005; Whitaker et al., 2007). Of additional interest is the comparison in reverse, where biological forms or materials are used to inspire and benefit human technologies, a field known as biomimetics, which includes the classic example of burdock fruits as models for Velcro fasteners (Vincent et al., 2006). Recently, there has been increased attention to the use of examples of functional plant morphology as inspiration for many biomimetic applications (Speck and Speck, 2021), and mechanisms of gametophyte dispersal in particular may serve as inspiration for various human purposes. For example, the moss peristome involved in spore dispersal is a superhydrophobic structure (extremely water repellent), whose material composition could be used in the development of human‐engineered superhydrophobic surfaces as nanocoating for water repellency or similar purposes (Barthlott et al., 2016).

Many of these dispersal mechanisms involve changes in the shape or size of structures in response to different stimuli, especially changes in hydration status or relative humidity. *Equisetum* elaters can be considered self‐propelled objects, whose movements are hygroscopic (driven by the absorption of water from the air), and could serve as models for self‐propelled objects for human use (Marmottant et al., 2013). Similarly, soft actuators are a class of materials that include polymers or other composites that change shape or size in response to stimuli (e.g., swelling or shrinking, twisting). Plants are noted as potential biological models for these soft actuators, including self‐burying seeds, pine cone seed scales, and carnivorous traps (Erb et al., 2013; Montero de Espinosa et al., 2017; Correa et al., 2020; Ren et al., 2021); however, little attention has been devoted to the biomaterials involved in gametophyte dispersal, which have similar shape‐changing properties. The multiple evolutionary mechanisms employed by plants provide different “solutions” to dispersing diaspores, and each of these mechanisms may also have the potential for human use in the fields of materials science, engineering, and medicine.

## CONCLUSIONS

High‐speed video and advanced microscopy have allowed scientists to understand some of the incredible biophysical mechanisms that have evolved in plants to disperse their gametophyte life stage. Rapid movements to assist in the dispersal of spores, asexual propagules, and pollen across land plant groups represent evolutionarily diverse systems for achieving dissemination of genetic materials. A comprehensive understanding of these mechanisms is critical for the ecology and evolution of land plants and can also contribute to advancing human technologies.

## AUTHOR CONTRIBUTIONS

N.M. and J.E. conceived of this review, read and summarized the literature, and wrote the first draft of the manuscript. N.P.P. took and processed the SEM images. J.E. and D.D.W. filmed the videos. All authors reviewed, edited, and approved the final manuscript before submission and publication.

## Supporting information


**Appendix S1.** Successive frames from a video of an exploding *Cornus canadensis* flower filmed at 1000 fps show that the film speed is not high enough to clearly capture the movements of the stamens, petals, and pollen. Filmed using a Reticon 256 × 256 high‐speed digital CCD camera, model MD4256C (EG&G, Gaithersburg, Maryland, USA). Numbers are milliseconds.

**Appendix S2.** Setup for filming high‐speed video of gemmae dispersal by gemmae‐dispersing splash cups of *Marchantia*.

**Video S1.** Insects visiting *Spiraea alba* (Rosaceae) filmed at 240 fps. File is 182 MB in size, 1080p, for a real‐time length of approximately 3.2 seconds. Insects were collecting nectar from the deep red nectar disc at the base of the flowers. Videos were filmed with the Sony camera using a tripod setup with post‐event triggering with a remote cable trigger. Playback is at 24 fps.

**Video S2.** Insects visiting *Spiraea alba* (Rosaceae) filmed at 480 fps. Files is 198 MB in size, 1080p, for a real‐time length of approximately 1.6 seconds. Insects were collecting nectar from the deep red nectar disc at the base of the flowers. Videos were filmed with the Sony camera using a tripod setup with post‐event triggering with a remote cable trigger. Playback is at 24 fps.

**Video S3.** Insects visiting *Spiraea alba* (Rosaceae) filmed at 960 fps. Files is 348 MB in size, 1080p, for a real‐time length of approximately 1.4 seconds. Insects were collecting nectar from the deep red nectar disc at the base of the flowers. Videos were filmed with the Sony camera using a tripod setup with post‐event triggering with a remote cable trigger. Playback is at 24 fps.
