# Supplementary material for: High‐speed video and plant ultrastructure define mechanisms of gametophyte dispersal
Source: Appl Plant Sci. 2022 Apr 20;10(2):e11463. doi: 10.1002/aps3.11463 (PMC9039801; doi:10.1002/aps3.11463)

DOI 10.1002/aps3.11463

APPENDIX S1. Successive frames from a video of an exploding *Cornus canadensis* flower filmed at 1000 fps show that the film speed is not high enough to clearly capture the movements of the stamens, petals, and pollen. Filmed using an Reticon 256 × 256 high-speed digital CCD camera, model MD4256C (EG&G, Gaithersburg, Maryland, USA). Numbers are milliseconds.

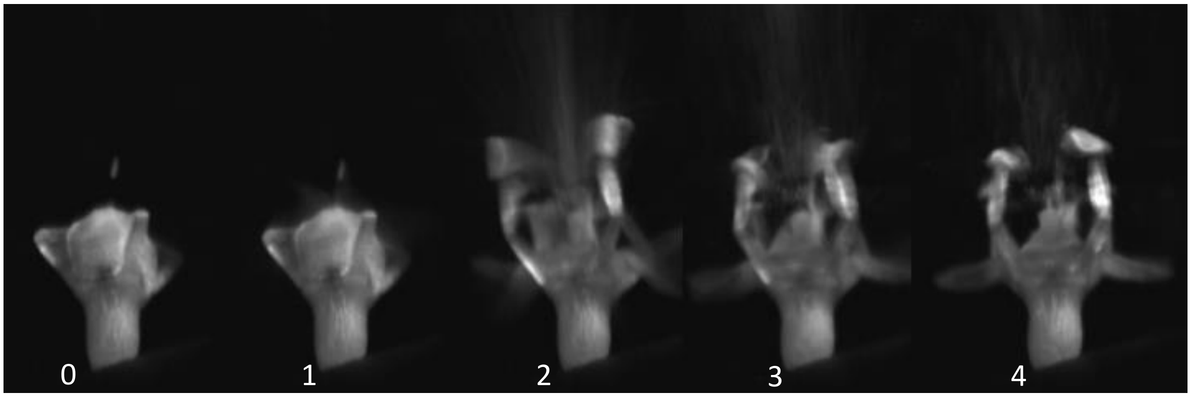

Supplement: Supplementary file 1 — Appendix S1. Successive frames from a video of an exploding Cornus canadensis flower filmed at 1000 fps show that the film speed is not high enough to clearly capture the movements of the stamens, petals, and pollen. Filmed using a Reticon 256 × 256 high‐speed digital CCD camera, model MD4256C (EG&G, Gaithersburg, Maryland, USA). Numbers are milliseconds. [file APS3-10-e11463-s005.pdf]
