# Supplementary material for: High‐speed video and plant ultrastructure define mechanisms of gametophyte dispersal
Source: Appl Plant Sci. 2022 Apr 20;10(2):e11463. doi: 10.1002/aps3.11463 (PMC9039801; doi:10.1002/aps3.11463)
Supplement: Supplementary file 2 — Appendix S2. Setup for filming high‐speed video of gemmae dispersal by gemmae‐dispersing splash cups of Marchantia. [file APS3-10-e11463-s004.pdf]

DOI 10.1002/aps3.11463

APPENDIX S2. Set up for filming high-speed video of gemmae dispersal by gemmae-dispersing splash cups of *Marchantia*.

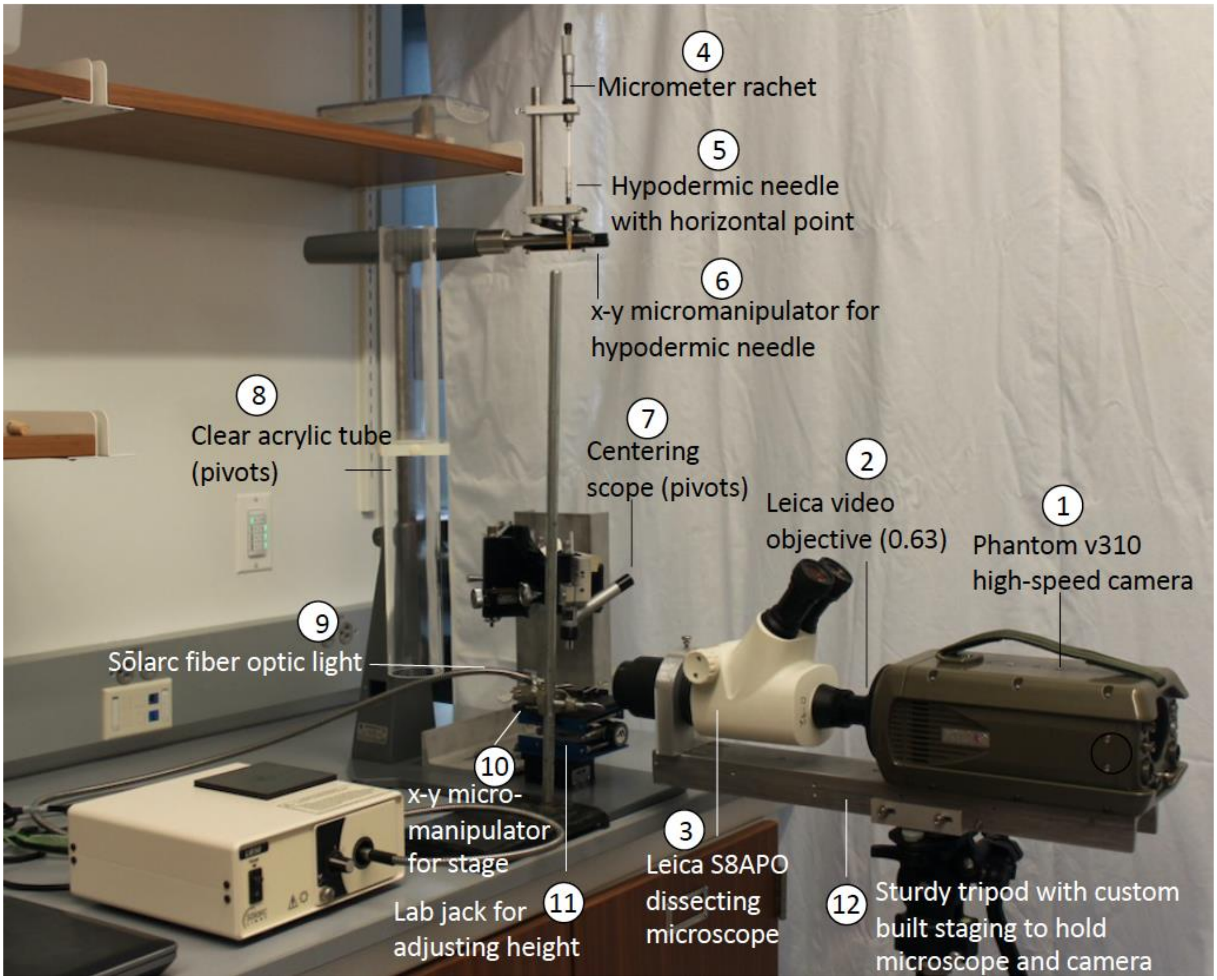

A key feature of this system is the ability to make fine adjustments in the x, y, and z planes for many of the components, using a dissecting microscope attached to an adjustable platform. Although this is specific for *Marchantia,* elements of this set up can be adopted for filming other systems. The studies in Table 1 provide other ideas for staging.

1. Phantom v310 high-speed camera connected to
2. a Leica video objective (0.63×), which connects through a camera port to
3. a Leica S8APO dissecting microscope.
4. A micrometer rachet that presses on
5. a hypodermic plunger to allow one drop to be dispensed at a time.
6. The hypodermic is held on a x-y micromanipulator to allow fine-scale adjustment of where the drop will hit.
7. A centering scope that allows alignment of the gemmae target (where the drop will hit). The centering scope pivots out of the way once the alignment is set.

DOI 10.1002/aps3.11463

8. Clear acrylic tube that can pivot into place during filming. This minimized air current interference with the falling drop.
9. Sōlarc fiber optic light. The position shown is backlit to visualize fluid mixing (with clear water in the cup and dyed water dropping from the hypodermic) in clear acrylic model splash cups.
10. x-y micromanipulator for the stage
11. Lab jack for adjusting the height of the stage.
12. A sturdy tripod with a hand crank for adjusting the height. A custom-built holder for the camera and microscope is affixed to the tripod. The holder allows the camera/microscope unit to slide forward and backward.
